# Routine Paediatric Sickle Cell Disease (SCD) Outpatient Care in a Rural Kenyan Hospital: Utilization and Costs

**DOI:** 10.1371/journal.pone.0061130

**Published:** 2013-04-09

**Authors:** Djesika D. Amendah, George Mukamah, Albert Komba, Carolyne Ndila, Thomas N. Williams

**Affiliations:** 1 African Population Health Research Center, Nairobi, Kenya; 2 Kenya Medical Research Institute (KEMRI) Centre for Geographic Medicine Research (Coast), Kilifi, Kenya; 3 INDEPTH Network of Demographic Surveillance Sites, Accra, Ghana; 4 Nuffield Department of Medicine, University of Oxford, Oxford, United Kingdom; 5 Global Network for Sickle Cell Disease, Toronto, Canada; Mahidol-Oxford Tropical Medicine Research Unit, Thailand

## Abstract

**Background:**

More than 70% of children with sickle cell disease (SCD) are born in sub-Saharan Africa where the prevalence at birth of this disease reaches 2% or higher in some selected areas. There is a dearth of knowledge on comprehensive care received by children with SCD in sub-Saharan Africa and its associated cost. Such knowledge is important for setting prevention and treatment priorities at national and international levels. This study focuses on routine care for children with SCD in an outpatient clinic of the Kilifi District Hospital, located in a rural area on the coast of Kenya.

**Objective:**

To estimate the per-patient costs for routine SCD outpatient care at a rural Kenyan hospital.

**Methods:**

We collected routine administrative and primary cost data from the SCD outpatient clinic and supporting departments at Kilifi District Hospital, Kenya. Costs were estimated by evaluating inputs - equipment, medication, supplies, building use, utility, and personnel - to reflect the cost of offering this service within an existing healthcare facility. Annual economic costs were similarly calculated based on input costs, prorated lifetime of equipment and appropriate discount rate. Sensitivity analyses evaluated these costs under different pay scales and different discount rate.

**Results:**

We estimated that the annual economic cost per patient attending the SCD clinic was USD 138 in 2010 with a range of USD 94 to USD 229.

**Conclusion:**

This study supplies the first published estimate of the cost of routine outpatient care for children born with SCD in sub-Saharan Africa. Our study provides policy makers with an indication of the potential future costs of maintaining specialist outpatient clinics for children living with SCD in similar contexts.

## Background

Haemoglobinopathies are the most common single gene disorder in the world [1] and 70% of children born with haemoglobinopathies actually have sickle cell disease (SCD) [2]. More than 70% of children with SCD are born in sub-Saharan Africa [1]–[3] where the prevalence at birth was estimated at 2% or higher in some selected areas in Africa [4], [5]. SCD is also the most common genetic disorder in many developed countries such as the United Kingdom and the United States of America, but with a much lower birth prevalence of around 1 in 2000 [6] and 1 in 2500 [7] respectively. High rates of child morbidity and mortality have long characterized the disease worldwide. More recently, however, the introduction of newborn screening, and other improvements in healthcare have all but eliminated SCD-related excess childhood mortality in developed countries. Numerous studies have been conducted in the developed countries on the treatment of children and adults with SCD [8]–[13], and their healthcare expenditures [14]–[16]. However, in sub-Saharan Africa, only a few studies have focused on the comprehensive care of children with SCD [17]–[19] and little is known on the cost of this care. Such knowledge is important for setting prevention and treatment priorities at national and international levels for at least two reasons. First, the high percentage of SCD gene carriers —which can reach up to 15% or 30% in many countries in sub-Saharan Africa — indicates that SCD will remain common in the foreseeable future [2]. Second, recent declines in infectious diseases and increased awareness of SCD imply that children born with the disease will increasingly survive the high mortality infancy period and will use the healthcare system.

This study aims at filling a gap in knowledge of the utilisation of medical services and the associated costs of the SCD outpatient clinic in Kilifi District Hospital (KDH), in coastal Kenya. Through this study we describe the routine utilization of the SCD outpatient clinic at KDH between 2003 and 2010 and estimate its costs from a provider perspective in 2010. Information presented here offers potentially useful insights to those aiming to provide such services in similar settings. This study represents the first step in estimating the total cost of the disease in the area, and the distribution of those costs by families and providers.

## Methods

### Study setting

The study was conducted at the Kilifi District Hospital, located on the Kenyan coast, which serves as a first-referral hospital to a population of approximately 500,000 people who live in Kilifi and the surrounding districts. KDH is the base for the Kenya Medical Research Institute (KEMRI)/Welcome Trust Research Programme where a diverse portfolio of research is conducted on a platform consisting of clinical surveillance of the paediatric wards linked to data from the Kilifi Health and Demographic Surveillance System (KHDSS). KHDSS monitors a population of approximately 250,000 living in an area of 891 km^2^ surrounding the Kilifi District Hospital that is home to around 80% of all children admitted to the paediatric wards [20]. KHDSS provides information on the resident population, its demographic characteristics, births, migration, deaths and cause of death.

Kilifi District Hospital is the only hospital in the Coast region in Kenya that provides specialist care to children with SCD. A dedicated SCD outpatient clinic at Kilifi District Hospital has existed for more than 20 years and data from this clinic have been computerised since 2003. Clinical information on patients with SCD served by this clinic in its first few years has been described earlier [19]. The SCD outpatient clinic is held weekly on Ministry of Health property by personnel employed by the Ministry or the research programme. Laboratory and pharmacy departments offering SCD-related services are staffed by the research programme.

### Costing perspective

We calculated economic costs of running the SCD outpatient clinic at KDH in 2010 from a provider perspective by aggregating the capital, recurrent and overhead cost of inputs. Theoretically, economic costs should reflect the opportunity costs of alternative use of the resources, but practically it is recommended to use market prices as proxy [21]. Capital costs represent assets used for more than a year such as equipment, furniture, and recurrent costs cover personnel, medication, supplies, building use, utilities, laboratory reagents, and equipment maintenance. The overhead included costs related to general services shared by other departments such as support personnel, administration, security, and other contracted services.

### Data collection and analysis

This study was based on primary data collected retrospectively from different departments within the research programme and from KDH between February and April 2011. SCD outpatient clinic utilization data came from the routine data management system and covered the years 2003 to 2010. The 2010 total cost was obtained by multiplying the SCD clinic inputs used by their unit cost. These input data were collected from the three sections of the outpatient clinic - medical consultation, pharmacy and laboratory – and the unit costs were obtained from the central administration, suppliers and local retailers.

The personnel data were collected in two stages: the number, and position (e.g. the number of clinical officers or laboratory technicians) came from their respective departments along with the time spent on SCD outpatient clinic work. Then, the yearly employer costs corresponding to the employees' grade and the mid-range steps were collected from the general administration. Employer costs included the salary and other benefits such as costs of employee health insurance and annual leave. Medications routinely received by children with SCD were stated by the clinical officer and confirmed by the pharmacist. Unit costs of medications came from the pharmacy and its procurement unit and the quantity from the data unit. The supplies costs were calculated similarly with quantities obtained from the specific department, and unit market price obtained from the procurement office or from retailers. The building use cost was included to reflect the economic cost of space utilization [22] and was based on the rental price of similar buildings in the hospital neighbourhood reflecting prevailing market price. The costs of utilities were based on estimates of average energy use and corresponding costs.

For economic cost of existing equipment, its 2010 replacement cost was obtained, spread out over useful years of life [21] and discounted to account for time preference for the present. We assumed that the equipment had no resale value at the end of its useful life, set conservatively based on the literature: 5 years for laboratory equipment and furniture, and 3 years for information technology equipment [23]. The Kenya short-term Treasury Bond interest for 3-year (5%) was used for discounting the information technology equipment and the 5-year rate (7%) for all other equipment. Assuming the annuity was payable in the beginning of the year, we used the rate for the years 2 and 4 plus 1, thus obtaining respectively 2.8594 and 4.3872 as the present value of USD 1 [21]. The cost of laboratory equipment maintenance was prorated to the share of its use for the SCD routine clinic.

The research programme provided an overhead cost estimate ranging from 15% to 20% of the program costs and this study used 17% of the total cost. The total cost based on the inputs was divided by the number of children seen in 2010 to obtain the mean cost per child.

All costs were collected in Kenya Shilling (KES) and converted to US Dollar(USD) using the average of the daily exchange rate during 2010, i.e., 1 USD = KES 79.261 [24]. We adapted a data collection tool developed for HIV/AIDS programs [22] written in Microsoft Excel (Seattle) to organize our data and analysed them using Stata/SE 10 (College Station).

### Sensitivity analysis

We assessed cost following changes in the main underlying conditions for sensitivity analysis. First, we used the Kenya Government personnel pay scale rather than that of the research programme. Second, we used a 3% discount rate as is common in the literature [23] thus a present value of annuity of USD 1 became 2.9135 and 4.7171 for 3 and 5 years respectively. Third, we used the range of 10% to 50% for overhead cost as has been calculated for comparable programs in sub-Saharan Africa and Kenya [25], [26]. Finally, we multiplied the personnel cost by two to reflect the possibility of two-day clinic considering the number of patients.

## Results

### Recruitment and routine care

The Kilifi SCD outpatient clinic is a specialist clinic for children with SCD. Children were referred to the Kilifi SCD outpatient clinic from three main avenues. Some children were referred from other departments within the hospital or different healthcare facilities in the region. Other children with SCD were identified through early life screening within the Kilifi Genetic Birth Cohort study described elsewhere [27]. Finally, siblings of index children with SCD were screened and if positive referred to this clinic.

**Figure 1:**
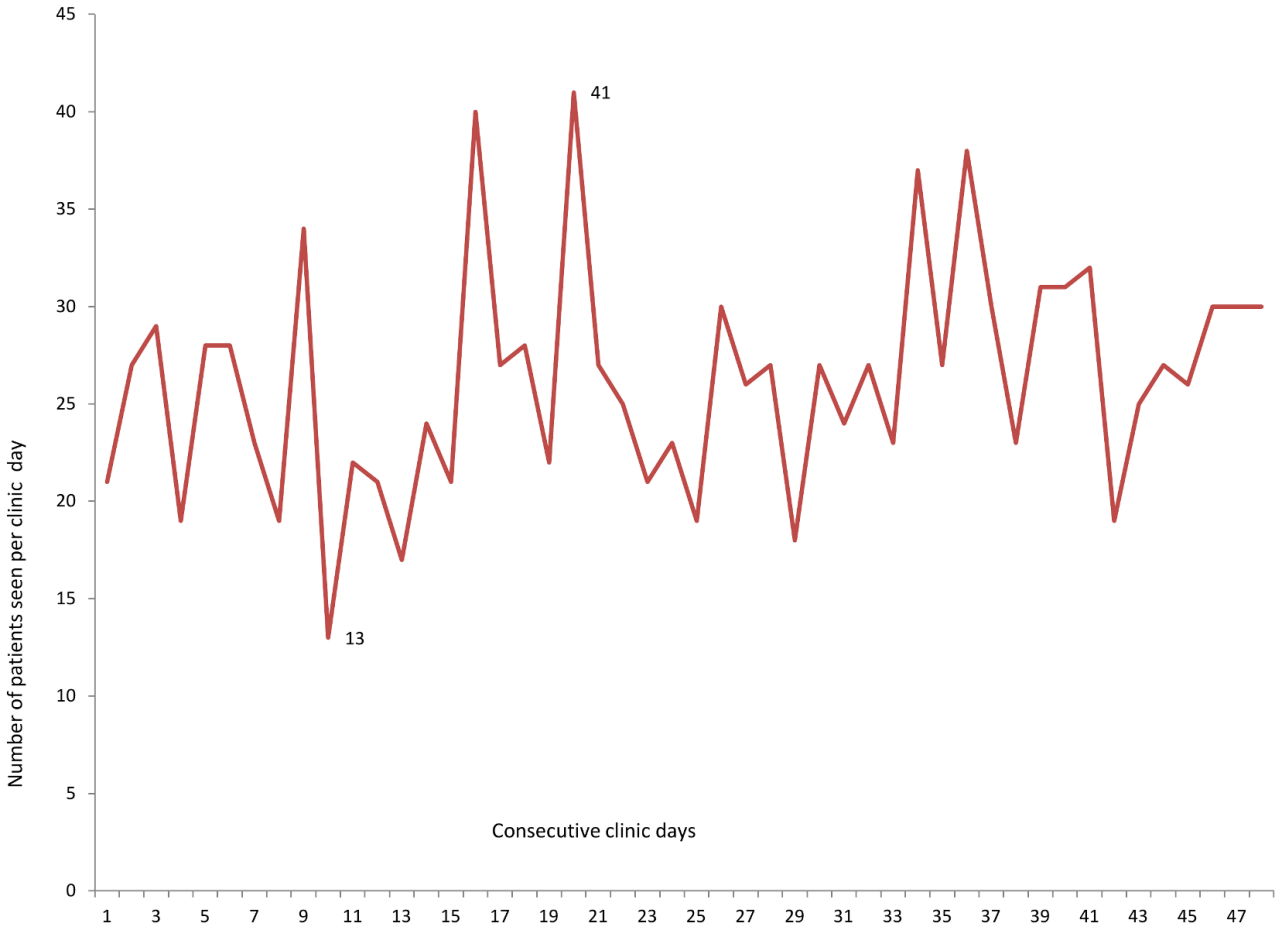
Number of patients seen on clinic days in 2010.

Table 1 indicates that the number of children attending this SCD clinic increased steadily from 119 in 2003 to 402 in 2010. Over this period, a higher proportion of patients (51%–60%) were male. The Wilcoxon test of equality of the numbers of girls and boys attending the clinic reject the null and the results is statistically significant (z = −2.524 p = 0.0116). The proportion of patients in all age groups attending the clinic increased over the years. In 2010, 38% of the children were 5 years or younger, 35% were aged 6 to 10 years, and 25% were between 11 and 18. The remaining 3% were older than 18 years. During 2010, the number of persons with SCD seen on clinic days varied from 13 to 41 (Figure 1).

**Table 1:** Number of children with SCD seen in outpatient clinic in Kilifi District Hospital by sex 2003 to 2010.

Year	Female	Male	Total
2003	48	71	119
2004	74	79	153
2005	94	106	200
2006	97	116	213
2007	125	145	270
2008	140	163	303
2009	164	186	350
2010	198	204	402

### Services offered by the SCD outpatient clinic

Children with SCD are scheduled for the SCD routine clinic on a quarterly basis although they are welcome to visit on a clinic day if they are sick. The SCD clinic runs once weekly thus occupying 20% of most of the personnel time. The SCD clinic is staffed by a clinical officer, a trained personnel in charge of data entry and anthropometric measurement, a phlebotomist, a medical record officer and a nurse from the general outpatient department. Each routine visit consists of a medical consultation, including the recording of anthropometric measurements and vital signs, a urine dipstick test for protein, glucose, nitrites and blood cells and a blood draw for additional laboratory tests. These laboratory tests include a blood film examination for malaria parasites, a full haemogram, a reticulocyte count and routine biochemistry (including plasma electrolytes, creatinine and liver function tests). A prescription for an anti-malarial drug (proguanil) and folic acid supplement are filled routinely for 3 months.

The SCD clinic exists alongside a general outpatient clinic where all children regardless of their SCD status receive paediatric care. The outpatient clinic offers emergency care as well. Furthermore, children with SCD who visit the hospital for any complaints on non-clinic days are seen in the general outpatient clinic.

### Economic cost

Table 2 indicates that in 2010, the average total economic cost per child was USD 138. The economic capital cost amounted to 2,774, the personnel cost 31,329, and the medication amounted to 7,154. The annual costs for supplies were estimated at USD 3,518. The personnel cost is the single most important component accounting for 56% of the total expenditures while capital cost accounted only for 5%. Equipment and building use could be considered as fixed cost as those inputs cannot be easily changed in the short run. Using that definition, fixed cost amount for 8% of the total cost of the clinic. Personnel cost was considered in this study as variable. Although changing the personnel would not be easy in the short run, adjustment was possible by increasing the number of day spent working in this clinic.

**Table 2:** Economic cost of routine Sickle Cell Disease Clinic, with the main method and sensitivity analyses in USD^1^.

Input	Main method: Personnel using KEMRI^2^ pay scale, 5% discount for IT equipment and 7% for others, 17% overhead	Sensitivity Analyses				
		Personnel using Kenya government pay scale and input cost method	Discount rate of 3% for all equipment	Overhead 10%	Overhead 50%	Two day clinic with same number of patients: Personnel cost multiplied by 2
**Capital Cost**						
**Equipment, furniture**	2,774	2,774	2,774	2,774	2,774	2,774
**Recurrent costs**						
**Personnel**	31,329	16,184	31,329	31,329	31,329	62,658
**Medications**	7,154	7,154	7,154	7,154	7,154	7,154
**Supplies**	3,518	3,518	3,518	3,518	3,518	3,518
**Utilities**	743	743	743	743	743	743
**Building use**	1,892	1,892	1,892	1,892	1,892	1,892
**Laboratory equipment maintenance**	8	8	8	8	8	8
**Subtotal excluding overhead**	47,419	32,274	47,228	47,419	47,419	78,748
**Overhead** 3	8,061	5,487	8,029	,742	23,709	13,387
**Total including overhead**	55,480	37,761	55,256	52,160	71,128	92,135
**Number of children**	402	402	402	402	402	402
**Total cost per child**	**138**	**94**	**137**	**130**	**177**	**229**

1 On average in 2010, all costs in US Dollars (1 USD = KES 79.261 Kenya Shillings).

2 KEMRI (Kenya Medical Research Institute)/Wellcome Trust Research Programme.

3 Overhead include support personnel, security, general administration and accounting services.

### Sensitivity analyses of economic costs

Using the Government of Kenya pay scale instead of KEMRI's, the average cost per child would have been USD 94. The personnel cost would have been USD 16,184, lower than the personnel cost of KEMRI by 48%. Using a 3% discount rate as opposed to 7% and 5% had insignificant effects on the economic cost–it dropped by USD 1 to USD 137 per child—as only the equipment was discounted. We also used 10% and 50% as alternate values for the percentage of overhead cost instead of 17% as in the base case. We obtained the range of USD 130 to USD 177. Finally, the personnel cost was doubled to account for the high attendance as up to 41 persons visited the clinic on some days in 2010. The variation provided the highest level of cost per patient in this study, USD 229.

## Discussion and Limitations

The number of patients seen per SCD outpatient clinic session increased steadily over the years of study and suggests an increase in the total cost with the number of patients. If the number of patients recruited kept growing, an increase in personnel and equipment would become necessary to maintain the quality of care. Although this SCD outpatient clinic was designed to treat children, 11 young adults aged 19 to 31 years attended it in 2010. Most of those young adults were long term clinic attendees while one attended for the first time at the age of 31 years. The presence of these young adults hints at the issues of transition that will need to be addressed as children grow into adulthood in the context of rare SCD specialized care. More boys than girls attended the SCD outpatient clinic over the years. Considering that SCD affects a similar proportion of boys and girls at birth, the higher proportion of males in this clinic sample leaves an open question about what happened to girls born with SCD.

We estimated that the overall annual economic cost per child for the provision of outpatient clinic care for children with SCD at KDH ranged from USD 94 to USD 229 in 2010. To our knowledge, ours is the first study that has attempted to quantify the cost of care provision to patients with SCD in an African hospital. While the current study focused on the costs relating to the provision of SCD routine outpatient care, we recognise that this only represents a small proportion of the total economic cost of SCD. Other SCD-related medical costs may cover outpatient items like antibiotic therapy, and emergency care. Note that pneumococcal vaccine is recommended to children with SCD. In Kenya, this vaccine is part of the general vaccine schedule for all children—contrary to the current practice in other countries in Africa—thus will not be specific to children with SCD. Other components of the total cost of SCD are inpatient care, non-medical direct cost (e.g. transportation) and indirect costs. Examples of indirect costs are missed educational opportunities for children with SCD, and loss of productivity of caregivers and adults with SCD. Analyses of the total costs attributable to SCD would be important in the context of African populations and health systems in which SCD might represent an increasing burden as economic and demographic transition associated with global political awareness lead to reduced child mortality. Such analyses will be the subject of future studies.

The provider-paid costs calculated in this study are attributable to SCD alone as other staples of paediatric healthcare like immunizations or anti-helminthic care etc. are not offered through the SCD clinic but through the general outpatient department. Without the research programme in Kilifi most of the costs of specialist care for SCD would be borne by families who can ill-afford it : 68.5% of the population of Kilifi District live in poverty [28], healthcare delivered in public facilities is not free for patients, and health insurance is rare in rural areas. To put costs calculated in this study into perspective, the Government of Kenya annual health expenditure per capita was about USD 14 in 2009 (the latest figures available) [29] or 18% of the lower bound of our estimates (i.e USD 94). Although provision of this routine care will help prevent morbidity and mortality, children with SCD in this population will remain more likely to be hospitalized than other children. Children with SCD account for 1.6% of all admissions to the paediatric wards at KDH [30] while the birth prevalence is only 0.8% in the area. In addition, US-based studies relying on annual insurance claims have indicated that approximately 40% of children with SCD incurred at least one hospitalization, about 50% of them visited the emergency department, and SCD-related medical care costs increased with age [15], [16]. While acknowledging the very different epidemiology and healthcare contexts between coastal Kenya and the United States, such figures along with the disproportionate share of SCD related hospitalizations at KDH suggest that the routine care cost are not the only SCD-related medical expenditures that those families would face. These numbers and facts give an insight into likely issues of adherence as they relate to the medical treatment for a genetic disease in a resource-constrained environment.

This study has a number of limitations. First, the routine data on clinic attendance does not distinguish between the scheduled routine care and the emergency visits so that we could not estimate if the latter contributed significantly to the high number of patients on some clinic days. We could not estimate the cost per visit either. Second, from a methodological perspective calculating the SCD clinic costs was simpler for separable and discreet inputs such as the number of pills given to a child, personnel time, and building use because the clinic was held once a week and occupied specific personnel and building space. However, this exercise was delicate for laboratory equipment and supplies and overhead because those components were not dedicated to children with SCD but served patients in multiple research programmes concurrently. Second, although unlikely to be substantial, the cost calculation did not include buffer stocks for medications and reagents. Finally, land purchase and building construction was purposefully excluded to reflect the cost of setting-up a SCD outpatient clinic within an existing health facility, as was the case at KDH. Such input costs could be substantial.

To account for the uncertainty related to the limitations, we conducted a sensitivity analysis varying personnel costs, overhead costs and equipment discounting rate one at a time. The data was sensitive to change in the personnel cost –which was the single highest percentage of the total costs– and that of the overhead. However, the results were robust to discount rate of equipment. Future studies will include a careful data collection and analysis of ingredients for overhead cost to allow apportioning the overhead based on the programme use.

## Conclusion

This study showed that between 2003 and 2010, the number of unique patients cared for in a provider-paid routine SCD outpatient clinic increased steadily. Such a rise was concurrent with a population based early life screening that raised awareness of the disease, a documented reduction in malaria and other infectious diseases in the area. A combination of these factors probably led to a decreased general infant mortality.

The estimated annual cost of routine outpatient care per child with SCD is USD138 with a range of USD 94 to USD 229 in 2010, with personnel costs representing the single largest component. Note that costs calculated in this study do include neither non-SCD outpatient care nor any type of inpatient cost etc. Thus the average total medical care cost of children with SCD is higher. Future studies will focus on the other components of the cost of Sickle Cell Disease. Nonetheless, these figures will provide African ministries of health and potential donors with an estimate of the cost for the provision of care to children living with this condition in similar contexts.
